# State-of-the-Art Review: Demyelinating Diseases in Indonesia

**DOI:** 10.1155/2021/1278503

**Published:** 2021-06-23

**Authors:** Hana Larassati, Riwanti Estiasari, Reyhan E. Yunus, Paul M. Parizel

**Affiliations:** ^1^Radiology Department, Dr. Cipto Mangunkusumo General Hospital, Jakarta, Indonesia; ^2^Faculty of Medicine Universitas Indonesia, Jakarta, Indonesia; ^3^Neurology Department, Dr. Cipto Mangunkusumo General Hospital, Jakarta, Indonesia; ^4^David Hartley Chair of Radiology, Royal Perth Hospital & University of Western Australia, Perth, Western Australia, Australia

## Abstract

Demyelinating diseases are more common in Indonesia than previously believed. However, it is still a challenge for a country such as Indonesia to implement the scientific medical advances, especially in the diagnostic process of demyelinating diseases, to achieve the best possible outcome for these groups of patients, within the constraints of what is socially, technologically, economically, and logistically achievable. In this review, we address the 4 major classes of demyelinating disease: multiple sclerosis (MS), neuromyelitis optica (NMO), anti-MOG-associated encephalomyelitis (MOG-EM), and acute disseminated encephalomyelitis (ADEM), and discuss their prevalence, demographics, clinical diagnosis workup, and imaging features in the Indonesian population, as well as the challenges we face in their diagnosis and therapeutic approach. We hope that this overview will lead to a better awareness of the spectrum of demyelinating diseases of the central nervous system in Indonesia.

## 1. Introduction

Indonesia is the largest archipelago in the world. Straddling equator, Indonesia is located on a crossroads between two oceans, the Pacific and the Indian Ocean, and bridges two continents, Asia and Australia. It is the most populous country in South East Asia and the fourth most populous in the world. Indonesia is a middle-income country, which, according to the World Health Organization (WHO), has a six times higher absolute burden of neurological disorders compared with high-income countries [1, 2].

Demyelinating diseases, predominantly multiple sclerosis (MS), are prevalent chronic inflammatory disorders of the central nervous system (CNS), with MS alone affecting more than 2 million people worldwide [3]. MS is most common in Europe, the United States, Canada, New Zealand, and Southern Australia; it is rarely found in tropical regions such as Asian countries [4]. The “Viking gene” hypothesis suggested that this uneven worldwide distribution was caused by a genetic predisposition linked to the Vikings, which explains the higher prevalence of MS in areas with many inhabitants of Scandinavian descent [5, 6]. To the best of our knowledge, the Vikings never visited Indonesia, even though they traveled far from their homelands across the Baltics, Western and Eastern Europe, the British Isles, the Mediterranean, and the North Atlantic, as far as Canada. Indonesia is a tropical country, and yet, we see a large number of patients with demyelinating diseases, not only in Caucasians descended from the Dutch colonialists but also in the native population.

In Indonesia, it is often challenging to ascertain the exact type of the demyelinating disease, because of the inability to perform a complete diagnostic workup, especially regarding the limited facilities for testing aquaporin 4 antibodies (AQP4-IgG) or anti-MOG antibodies. Dr. Cipto Mangunkusumo National Public Hospital is the national tertiary referral center in Indonesia, examining patients suspected of demyelinating diseases from all over the country. From the departments of neuroradiology and neurology, Dr. Cipto Mangunkusumo National Public Hospital, this review addresses the 4 major classes of demyelinating disease: multiple sclerosis, neuromyelitis optica (NMO), anti-MOG-associated encephalomyelitis (MOG-EM), and acute disseminated encephalomyelitis (ADEM), and discusses their prevalence, demographics, clinical diagnosis workup, and imaging features in the Indonesian population. Information regarding the healthcare system and cost and coverage for imaging and laboratory testing, as well as medications, was obtained from local contacts to relevant agencies and laboratories in Indonesia.

## 2. Demyelinating Diseases

### 2.1. Multiple Sclerosis (MS)

Data from the Multiple Sclerosis Federation Atlas of MS indicated that there are currently 160 cases of MS in Indonesia in 2020 [7]. However, the Global Burden of Disease Study 2016 (GBD 2016) estimated the number of MS cases in Indonesia to be approximately 7056 cases [8]. Data gathered from our center, “Dr. Cipto Mangunkusumo General Hospital,” the national referral hospital of Indonesia located in Jakarta, indicated that there were 56 new cases of MS between 2015 and 2020. The number of cases seemed to be increasing annually, with 1-2 more new cases reported per year. The increase of MS cases in Indonesia has also been documented in the GBD 2016, which estimated 37.1% increase from 1990 to 2016 [8].

MS is a chronic inflammatory disease and clinically classified into 4 types of disease courses: clinically isolated syndrome (CIS), relapsing-remitting MS (RRMS), secondary progressive MS (SPMS), and primary progressive MS (PPMS). Globally, MS is often seen in young adults, more predominant in female population, and known to cause both physical and cognitive disability. In Indonesia, MS affects patients in various age brackets, with cases occurring in pediatric up to geriatric age groups, though there is a clear predominance in young adults. The median age of disease onset of our MS patients is 25 years old (range between 15 and 48 years old). Indonesian MS patients are predominantly females, with a 4 : 1 ratio to males, consistent with global demographic reports. MS patients in our center came from various Indonesian ethnic groups, namely, Javanese, Sundanese, Minang, Batak, Palembang, Chinese, Manado, and Arab. The Javanese ethnic group made up the largest proportion of MS patients in our center (36.4%), followed by Chinese descents (24.2%). However, none of our reported patients were of the easternmost Indonesian ethnic groups, i.e., Moluccan, or ethnic groups from the Papua regions. This is likely due to the geographical location of our center, since Jakarta is located in the Western part of Java and is relatively distant from the islands in the easternmost regions of Indonesia. The most common MS type in Indonesia is RRMS (76.8%), followed by SPMS (19.6%), which is consistent with the global epidemiological data.

According to present guidelines, MS diagnosis is based on the McDonald criteria 2010 [9]. The criteria rely on clinical findings and supporting evidence from auxiliary tests, such as magnetic resonance imaging (MRI) of the brain and spinal cord, as well as cerebrospinal fluid (CSF) analysis. In our center, patients presenting with a neurological deficit that could be compatible with MS typically undergo blood studies to help exclude other conditions, such as antinuclear antibody- (ANA-) associated autoimmune disorders (to exclude the possibility of other autoimmune disease), infections, or endocrine abnormalities. Lumbar puncture is routinely performed, even though not all give consent to undergo the procedure. In our center, there are, on average, 4 to 5 patients who underwent lumbar puncture per month. Moreover, though the oligoclonal band screen facility is available, this test is not covered by the national health insurance and is considered expensive.

One of the main differential diagnoses of MS is neuromyelitis optica (NMO), which is typically differentiated from MS by the presence of serum aquaporin 4 antibodies (AQP4-IgG). The cost for AQP4-IgG testing is expensive for the patient, and therefore, to our regret, we cannot routinely perform this test in patients suspected of MS.

#### 2.1.1. Imaging

In our center, MRI examinations are performed at a magnetic field strength of 1.5 T, with 5 mm slice thickness. The protocol for brain MRI in suspected MS cases consists of axial and sagittal T1WI without contrast; axial, sagittal, and coronal T1WI with contrast; axial and coronal T2WI; axial FLAIR; axial T2∗ gradient-echo (GRE); and axial DWI. Three-dimensional thin-slice sequences, such as 3D T2-FLAIR or 3D T1WI MPRAGE, are not routinely used due to the longer examination time required. Baseline and follow-up examinations are both done with the same protocol. For spinal cord MRI, we use axial and sagittal T1WI with and without contrast, sagittal conventional T2WI, and axial and sagittal T2WI with turbo inversion recovery magnitude (TIRM) sequence. However, in our center, spinal cord MR is not automatically alongside brain MR in suspected MS cases. This is largely due to the policy of national health insurance in Indonesia, which does not always cover multiple MR examinations in a single appointment.

According to the Magnetic Resonance Imaging in MS (MAGNIMS) consensus, brain MRI examinations in suspected or confirmed MS patients should consist of a multisequence MRI, performed at a magnetic field strength of at least 1.5 T (preferably 3.0 T) with a maximum slice thickness of 3 mm and an in-plane spatial resolution of 1 × 1 mm [10, 11]. A comparison between our standard protocol and MAGNIMS recommendation is described in Table 1.

MS lesion on MRI is defined as an area of focal hyperintensity on a T2-weighted (T2, T2-FLAIR, or similar) or proton density-weighted sequence. Characteristic locations for MS are the periventricular, juxtacortical, and infratentorial regions and the spinal cord. In patients suspected of MS, the imaging criteria for diagnosis are based on the demonstration of disease dissemination in time and space on dual-echo and contrast-enhanced T1WI. There are several MRI criteria for dissemination in space and time for MS, including the MAGNIMS criteria, criteria proposed by Swanton et al., and revised international panel criteria [12, 13]. In Indonesia, imaging criteria for MS diagnosis follow the MAGNIMS consensus.

**Figure 1:**
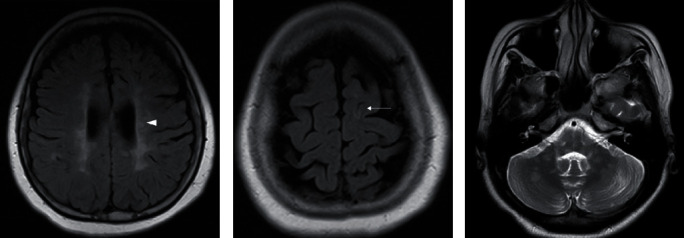
MRI of two different MS patients in our center: (a, b) a 30-year-old female with SPMS, disease duration of 10 years, showing periventricular (arrowhead) and juxtacortical (arrow) lesions on axial FLAIR images and (c) 27-year-old female with RRMS, disease duration of 3 years, showing infratentorial lesions on axial T2WI.

**Figure 2:**
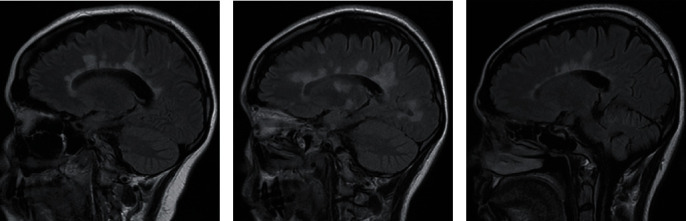
Sagittal FLAIR images from different MS patients in our center: (a) a 28-year-old female with RRMS, disease duration of 13 years; (b) a 29-year-old female with RRMS, disease duration of 1 year; (c) a 38-year-old female with RRMS, disease duration of 12 years, showing periventricular lesions with the characteristic “Dawson finger” appearance, indicating extension along the medullary veins.

**Figure 3:**
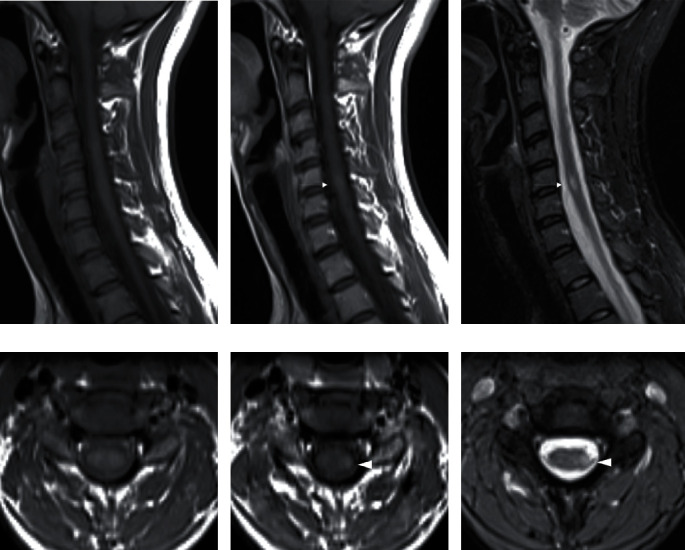
Cervical spinal cord MRI of an MS patient in our center, a 29-year-old female with RRMS: (a) sagittal unenhanced T1WI, (b) contrast-enhanced T1WI, and (c) T2WI TIRM revealing a short-segment, mildly contrast-enhancing spinal cord lesion at the level of C5-6 (arrows). Corresponding axial images showing the same lesion (arrowheads) on (d) unenhanced T1WI, (e) contrast-enhanced T1WI and (f) T2WI TIRM.

On brain MRI of Indonesian MS patients, we found multiple hyperintense lesions on T2-weighted (T2 or T2-FLAIR) sequence, most commonly in the periventricular region (93.1% of patients), followed by other characteristic areas, such as juxtacortical (82.7%) and infratentorial (69%) (Figure 1). Infratentorial lesions are better visualized in T2WI compared to FLAIR, due to the diminished contrast of posterior fossa lesions in the FLAIR sequence [14]. Most patients (69%) with periventricular lesions have extending lesions along the axis of medullary veins (“Dawson fingers”) (Figure 2). Cortical lesions are less frequently observed (51.7%). Lesions in Indonesian MS patients are often multiple, ovoid in shape, and small. On spine MRI, most lesions in our patients involved the dorsal columns of the spinal cord. The spinal cord lesions are more frequently seen in the cervical region and have short craniocaudal diameter (covering less than two vertebral segments), and on axial images, they are located in the peripheral portions of the spinal cord (Figure 3). In terms of location and lesion characteristics, we observe similar imaging findings between MS patients in the Indonesian population and other population described in the literature [13]. However, contrast enhancement of the lesions was rarely seen in our patients. Follow-up MRI examinations in Indonesian MS patients were done according to patient's individual clinical course, most commonly once every 6-12 months.

Recently, advanced imaging technique, such as DTI, gained emphasis in the evaluation of MS. Previous studies observed diffusion changes in the normal-appearing brain and spinal cord parenchyma of MS patients, showing the usefulness of diffusion MRI in evaluating microstructural changes, even when lesions are not detected on conventional MRI [15, 16]. Furthermore, diffusion changes are correlated with clinical aspects, such as cognitive dysfunction [17]. In our center, we recently conduct a study using ADC and FA value to evaluate the normal-appearing white matter (NAWM) of MS patients and found a significant correlation between the ADC and FA of corpus callosum NAWM with cerebral volume and, to a lesser degree, clinical disability [18].

### 2.2. Neuromyelitis Optica (NMO)

NMO, or Devic's disease, is an inflammatory disease of the central nervous system, presenting as either monophasic or recurrent attack of optic nerves (optic neuritis) and spinal cord (myelitis). A new term “NMO Spectrum Disorder (NMOSD)”, as well as new diagnostic criteria, has been developed since the discovery of specific autoantibody response against astrocyte water channel aquaporin 4 (AQP4). NMOSD prevalence in the global population ranges from 0.0003% to 0.0044%. It was suggested that NMOSD is more prevalent in Asian countries and has ethnic predilection for non-Caucasians, due to higher sun exposure and lower vitamin D levels in Asian population [19–21]. However, reports from various countries showed no clear latitude gradient in NMOSD prevalence, and low vitamin D levels in NMOSD were only incidentally reported [22]; thus, this theory is not proven. Recent studies in the Han Chinese population showed an association between NMOSD and GTF2I gene polymorphism. This gene plays a role in humoral immunity regulation; hence, polymorphism in the GTF21 gene may predispose patient to autoimmunity and possibly explains why NMOSD has strong association with other autoimmune diseases [23].

In Indonesia, we found only two studies on NMOSD, one of which reported 19 NMOSD cases in Indonesia [24, 25]. It has to be noted that these studies may not represent the actual burden of disease, and combined with the scarcity of AQP4-IgG testing facilities, it is likely that we underestimate the prevalence of NMOSD in Indonesia. There has been a long history of migration from China to Indonesia, and currently, there are considerable numbers of Chinese descents living in Indonesia. However, to date, there are no studies relating to the genetic profile of NMOSD patients in Indonesia, both in Chinese descents and other ethnicities. The reported NMOSD patients in Indonesia were predominantly female with a mean age of 35.5 ± 11.4 years old, similar with global reports that document female predominance in NMOSD. The majority of Indonesian NMOSD patients (12 out of 15) had positive AQP4-IgG, with higher relapse rate compared to seronegative patients [24]. This is similar to other studies that showed NMOSD with negative AQP4-IgG are more frequently monophasic [19]. Once again, it has to be noted that Indonesia has only 3 AQP4-IgG testing facilities and that this test is not covered by the national health insurance. The 2015 International Panel for NMO Diagnosis (IPND) recommends repeating serologic test for relapsing AQP4-IgG-seronegative NMOSD, because patients may convert to become seropositive over time [19, 21]. However, repeated testing is rarely done in Indonesian NMOSD patients because there are very few AQP4-IgG testing facilities. The lack of testing laboratories can be attributed to the high cost of the test and may hinder testing for patients in remote areas in Indonesia, as well as repeated testing for patients with negative AQP4-IgG. This problem is not unique to Indonesia, since multinational studies on availability and affordability of NMO diagnostic testing showed that AQP4-IgG testing facilities are only available in 38% of low-income countries, indicating a gap in access to diagnostic testing for NMO [26].

#### 2.2.1. Imaging

**Figure 4:**
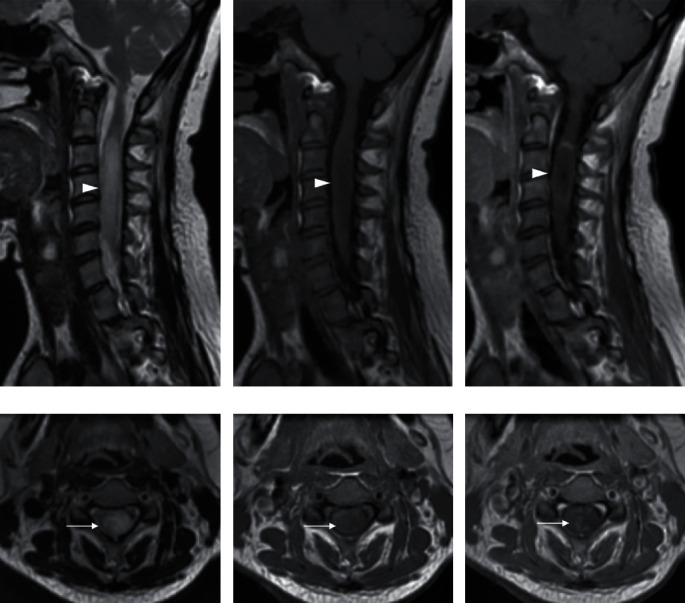
Cervical spinal cord MRI of an NMO patient in our center, a 32-year-old female, with left-sided hemiparesis for two weeks, showing (a) T2WI-hyperintense longitudinally extensive myelitis, spanning across more than 3 vertebral segments; (b) hypointense on unenhanced T1WI; and (c) with peripheral enhancement on contrast-enhanced T1WI (arrowheads). Corresponding axial (d) T2WI, (e) unenhanced T1WI, and (f) contrast-enhanced T1WI, showing the involvement of more than 50% of the cord surface area (arrows).

**Figure 5:**
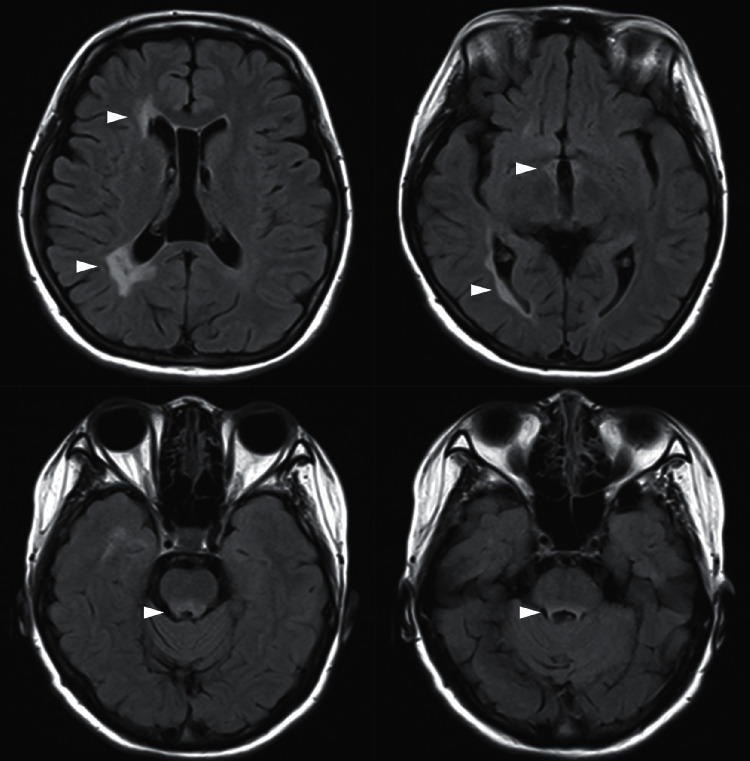
Axial FLAIR images from a 28-year-old female with NMO, disease duration of 2 years, with bilateral visual impairment and general weakness, showing high signal white matter lesions in typical periependymal regions (arrowhead).

MRI is the main modality for radiologic evaluation of NMOSD. On spine MRI, we often see lesions in the cervical spinal cord in Indonesian NMOSD patients (37.5%). We observe hyperintense T2WI and hypointense T1WI lesions affecting more than three contiguous vertebral segments of the spinal cord (“longitudinally extensive transverse myelitis”). On axial images, the lesions predominantly involve the central cord and cover more than 50% of the cord surface area (transversally extensive). Contrast enhancement of the lesions with peripheral ring-like pattern is also seen (Figure 4). On brain MRI, Indonesian NMOSD patients have confluent, asymmetrically distributed hyperintense T2WI/FLAIR lesions, mostly in periependymal regions (43.7%) (Figure 5). AQP4 is consistently expressed in periependymal regions, making them the typical locations for NMOSD lesions. Indonesian NMOSD patients show typical imaging features of NMOSD lesions on both spine and brain MRI, similar to other NMOSD population described in the literature [19, 27].

Optic nerve lesions were demonstrated in both seropositive and seronegative groups of Indonesian NMOSD patients, while longitudinally extensive transverse myelitis (LETM) was found predominantly in AQP4-IgG-seropositive patients. Multiple T2 high signal brain lesions were only found in AQP4-IgG-seropositive patients [24]. In our center, MRI examinations of the brain and spinal cord for suspected NMO cases are performed with the same standard imaging protocol as described in Table 1. Accurate assessment of the optic nerves is sometimes difficult due to the lack of fat-saturated sequences and the 5 mm slice thickness.

### 2.3. MOG-IgG-Associated Encephalomyelitis (MOG-EM)

The role of immunoglobulin G serum antibody to myelin oligodendrocyte glycoprotein (MOG-IgG), a glycoprotein located on the myelin surface of the CNS, has been a focus of neuroinflammatory research in recent years [28]. MOG-IgG is now considered as a separate disease entity from both MS and NMO, referred to as MOG-IgG-associated encephalomyelitis (MOG-EM). It has substantial overlap with other demyelinating diseases in terms of clinical and radiological features; thus, the diagnosis should always be accompanied by MOG-IgG testing. The proposed diagnostic criteria for MOG-EM are based on a combination of [1] monophasic or relapsing acute optic neuritis, myelitis, brainstem encephalitis, or encephalitis, or any combination thereof; [2] MRI or electrophysiological (visual evoked potentials in patients with isolated optic neuritis) findings compatible with CNS demyelination; and [3] seropositivity for MOG-IgG as detected by means of a cell-based assay employing full-length human MOG as target antigen [28, 29].

In Indonesia, as yet, there have been no reports on MOG-EM cases. Epidemiological data is currently still unavailable, given the recent discovery of this entity. We have had 3 suspected cases of MOG-EM in our center, but in none of the patients the diagnosis was confirmed. The current absence of reports and the likely underdiagnosis of this condition are caused by the unavailability of the MOG-IgG serological test in Indonesia. Currently, our center has the only MOG-IgG serological testing facility in Indonesia and is still reserved strictly for research purposes.

#### 2.3.1. Imaging

Imaging, particularly MRI, plays an important role in the diagnosis of MOG-EM. The diagnostic criteria for MOG-EM require the patient to have MRI findings compatible with CNS demyelination, not specific to MOG-EM, to be diagnosed with this entity. This is mainly due to the overlap of MOG-EM MRI findings with other demyelinating diseases, notably MS and NMO. However, recent studies have shown that MRI can also be used to separate MOG-EM from MS and NMO. In approximately 1/3 cases of MOG-EM, there was optic neuritis with inflammation and enhancement of the perioptic nerve sheath, partly extending into the surrounding orbital fat. Furthermore, the optic nerve involvement was bilateral in 25% cases, more anterior, stretched, and edematous, with extended inflammation, differentiating it from the features of MS or NMO [30]. Brain lesions in MOG-EM are often confined to the pons and adjacent to the fourth ventricle [31]. We currently do not have the data regarding imaging features of Indonesian MOG-EM patients, since there are still no confirmed cases in Indonesia.

### 2.4. Acute Disseminated Encephalomyelitis (ADEM)

Acute disseminated encephalomyelitis (ADEM) is an immune-mediated inflammatory demyelinating disorder of the central nervous system which is commonly preceded by an infection. ADEM mainly affects children, with an estimated incidence of 0.4/100 000/year among persons under the age of 20; the mean age at presentation ranges from 5 to 8 years. ADEM has little to no gender predilection, in contradistinction to the female predominance in MS and NMOSD [32, 33]. There is no published reports regarding the prevalence of ADEM in Indonesia. However, a retrospective search of the PACS in our radiology department revealed 14 patients with ADEM between 2015 and 2021. Patients' age ranged from 1 to 38 years old (median 11 years old); they are predominantly male, with 4 : 1 male to female ratio. This pronounced male predominance is not in accordance with the literature, which reported equal incidence in both sexes or only a slight male predominance [32, 34, 35]. Diagnosis is also more complicated in the adult patients suspected of ADEM. Patients in this demographic group may have several possible differential diagnoses, especially those which presented with clinical features atypical for ADEM, such as persistent meningeal signs or headache, and stroke-like events. Primary CNS vasculitis, secondary CNS vasculitis (e.g., Behçet disease and CNS lupus), and infectious encephalitis were among the differentials. These entities may also show similar appearance on imaging studies. However, extensive testing for these differentials, including screening test for infectious agents, may assist in the diagnosis [34].

ADEM usually has rapidly progressive monophasic course with favorable long-term prognosis [34, 36, 37]. The IPMSSG proposed diagnostic criteria for pediatric ADEM, which were updated in 2013 and required both clinical and imaging criteria on brain MRI [37, 38]. In our hospital, contrast-enhanced brain CT is usually requested in the emergency department to exclude other causes, such as infection or hemorrhage. After exclusion of other acute intracranial abnormalities, contrast-enhanced brain MRI is requested. A diagnosis of ADEM is then made according to the proposed diagnostic criteria. Serum AQP4-IgG and anti-MOG antibodies are not routinely examined because of the lack of testing facilities and because they are not covered by the national health insurance. Another hindrance for ADEM diagnosis in our center is the limited operational hours and patient capacity of MRI facility; thus, it is not available for patients in the emergency department and once requested, the waiting list can be long (sometimes months). Additionally, there are relatively few MRI facilities in Indonesia and the referral process to a hospital with MRI facility can be long. Therefore, patients with suspected ADEM can have normal brain MRI when it is finally done, because the acute phase has already passed. These problems potentially cause underestimation of ADEM prevalence in the Indonesian population.

#### 2.4.1. Imaging

Head CT is usually the first imaging modality requested in the acute setting. In our center, CT is usually done in the emergency department to exclude other causes, especially infections, due to the relatively high incidence of CNS infections in our population. However, in the diagnosis of ADEM, CT is typically not contributive unless large lesions are present, which appear slightly hypodense compared to the surrounding brain parenchyma [39].

**Figure 6:**
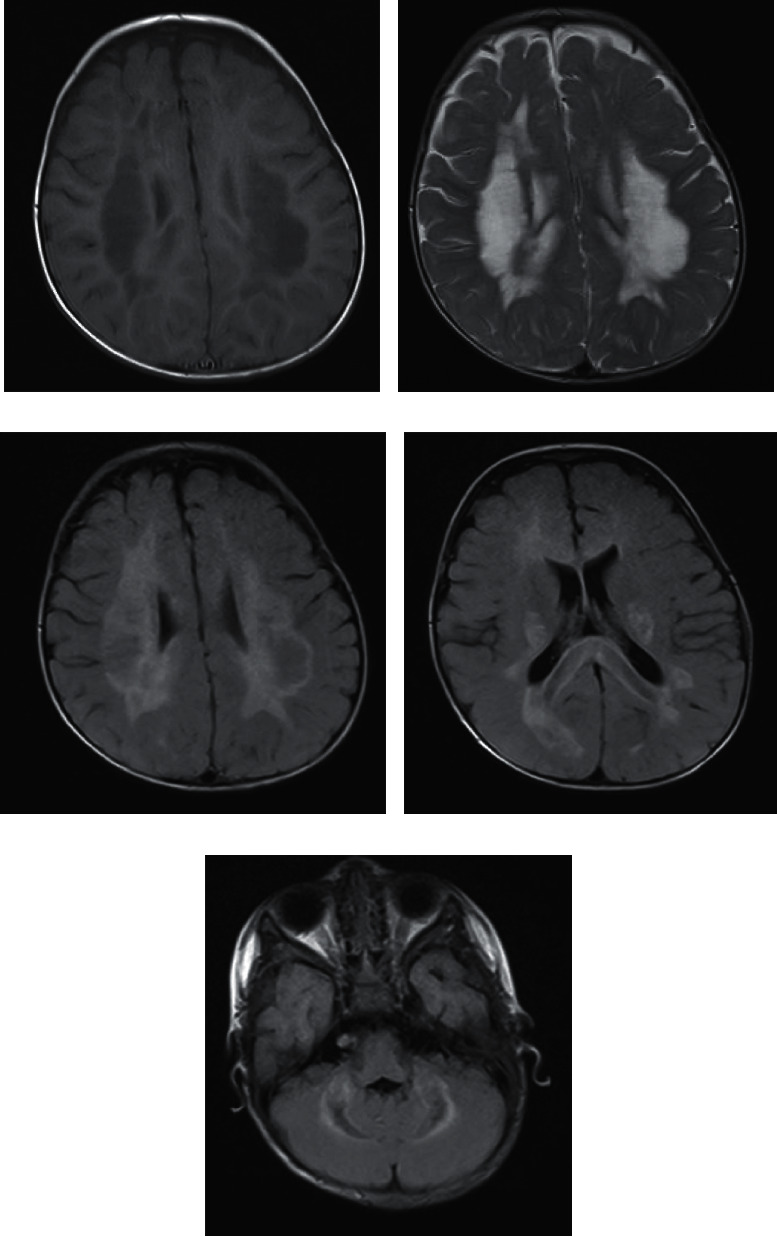
Axial brain MRI of a 1-year-old ADEM patient, who presented with stiffness of bilateral upper and lower extremities, with history of recent upper respiratory tract infection, showing large, bilateral, diffuse lesions, predominantly affecting the deep white matter, the corpus callosum, and bilateral cerebellar peduncles. The lesions are hypointense on unenhanced T1WI (a), hyperintense on T2WI (b), and partially suppressed on FLAIR (c–e).

**Figure 7:**
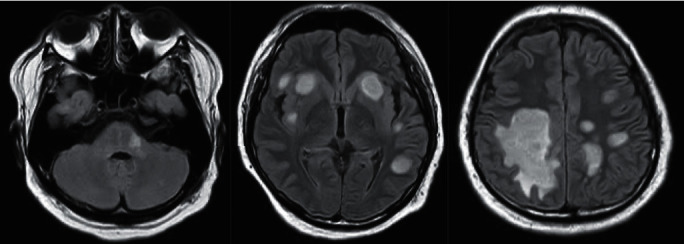
Axial FLAIR brain MRI of an 18-year-old male with ADEM, who presented with tetraparesis, predominantly on the left side. There were multiple hyperintense lesions on the left cerebellar peduncle, left cerebellum, and bilateral cerebral white matter.

Brain MRI is obtained in the presence of suggestive clinical findings. On brain MRI, we observed disseminated, bilateral, asymmetrical white matter lesions of T2WI increased signal intensity in Indonesian patients with suspected ADEM. Lesions also affect infratentorial regions, such as cerebellar peduncles. The lesions are poorly demarcated with variable size and relatively sparing the periventricular white matter, which are characteristic features of ADEM [40]. In terms of lesion characteristics, both pediatric (Figure 6) and adult (Figure 7) Indonesian ADEM patients had relatively similar lesion characteristics. We observed similar imaging findings between ADEM patients in the Indonesian population and other population described in the literature. However, there were a few cases with corpus callosum involvement, which is rarely seen according to the literature [39, 40].

The MRI appearance of ADEM has some overlapping features with its differential diagnosis, such as MS and CNS vasculitis. Periventricular sparing and the absence of “Dawson finger” appearance differentiate ADEM from MS. [34] Differentiating ADEM from CNS vasculitis on imaging alone may be difficult; however, angiography may show abnormal vessels in vasculitis. Secondary CNS vasculitis such as Behçet disease and CNS lupus would have other organ involvements outside the CNS [41].

We do not have a specific MRI protocol for patients suspected with ADEM, and brain MRI examinations were performed using our standard protocol (Table 1). Gadolinium-based contrast agents were rarely given to pediatric patients aged 2 years old or younger, and spinal cord MRI was rarely performed at the same time as brain MRI, due to the national health insurance policy which does not always cover multiple MRI examinations in one appointment.

## 3. Discussion and Conclusion

The obvious and most important conclusion is that demyelinating diseases are more common in Indonesia than previously believed. Moreover, demyelinating diseases are not limited to Caucasians but also affect other ethnicities in Indonesia, as described in Table 2. Another important thing to be reported is the high proportion of Indonesian Chinese descent ethnicity in MS and NMO (24.2% in MS and 16.7% in NMO). This is a relatively large proportion, since Indonesian Chinese descents only made up 1.2% of the Indonesian total population [42]. The distribution of other demographic characteristics such as sex and age of demyelinating disease patients in Indonesia is somewhat consistent with that reported in literature, except for ADEM, which showed pronounced male predominance, as seen in Table 3. Larger, nationwide data set is required to conclude a more accurate demographic pattern in the future. However, in the meantime, data gathered in Dr. Cipto Mangunkusumo General Hospital may provide a representative for the demyelinating disease patients in Indonesia, since it is a tertiary referral center which admits patients from all over the country.

The Dr. Cipto Mangunkusumo General Hospital is a 927-bed academic center located in Jakarta, the capital city of Indonesia, and it is the national referral hospital of Indonesia. Our neurology service has 26 consultant neurologists, with one neurologist subspecializing in inflammatory and immune-related disorders. The radiology department has 22 attending consultant radiologists, including 4 radiologists in the neuroradiology division. There are 2 fully operational 1.5 T MRI units, which each examine 30-40 patients per day. This patient load limits the examination time assigned for each patient, which means that we cannot always perform additional or time-consuming sequences, as recommended by the literature. Average waiting time for MRI examinations in our department varies between 2 and 6 weeks, depending on the patient load at the specific time. This rather long waiting time implies that, in most cases, the acute phase of the disease is missed; therefore, acute imaging findings such as breakdown of the blood-brain barrier (contrast enhancement) in MS lesions are rarely observed. However, in general, MRI appearances of demyelinating disease patients in our population are in accordance with the literature.

**Figure 8:**
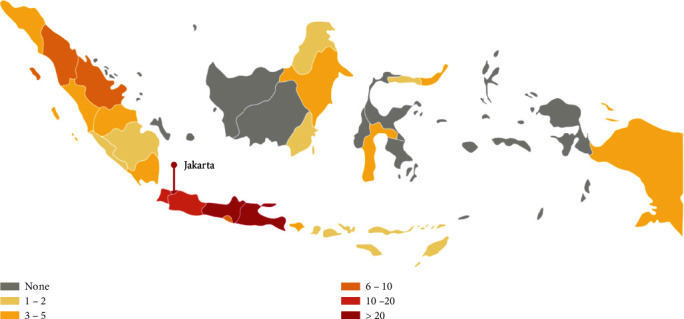
Distribution map of MRI facilities in Indonesia, showing the total number of MRI unit in each province [43].

In the diagnostic workup, international consensus and guidelines are implemented as much as possible. However, Indonesia faces serious obstacles in the implementation, which can result in a suboptimal diagnostic workup and may lead to underdiagnosis of certain conditions. There are 195 MRI units in total in Indonesia, which are spread across 25 provinces (out of 34 provinces) and which cover 266 million people of Indonesian population. This results in a ratio of one MRI unit per 1.3 million people. Moreover, 9 out of 34 provinces do not have MRI service at all, which may limit the diagnostic ability in these provinces. Patients in need of MRI examination must be referred to hospitals in another provinces, which are sometimes located in a different island and require a significant travel cost (Figure 8). MRI examinations of patients with demyelinating disease are covered by the national health insurance, but this usually includes only one examination per appointment, meaning that it is not always possible to perform MRI of the brain and of the spine in one session (unless the patient would be willing to pay out of pocket). Indonesia is lacking in the availability of testing kits which are needed to identify the subgroup of demyelinating disease (MS, NMO, MOG-EM, and ADEM). AQP4-IgG testing is available in only 3 facilities in Indonesia. The laboratory in our center is reserved strictly for research purposes (and does not perform routine diagnostic procedures), the second is a private laboratory (which transfers specimens to another laboratory in the United States), and the third one is located in a government hospital; all of these facilities are located in Jakarta. MOG-IgG testing is available in our center but is currently reserved for research purposes only. More importantly, testing for AQP4-IgG, MOG-IgG, and CSF oligoclonal bands is not covered by the national health insurance in Indonesia; AQP4-IgG testing costs 98 to 243 USD, and CSF oligoclonal band testing costs 165 to 341 USD. This implies that these relatively expensive tests are often unaffordable for most patients, which results in a very low number of tests being performed (3-4 patients for AQP4-IgG and 0-1 patient for CSF oligoclonal band per month). Other diagnostic facilities, such as visual evoked potential (VEP), optical coherence tomography (OCT), and fundus photography, are available in our center. VEP examination facilities are widely available and covered by the national health insurance. Albeit the utilization of a range of diagnostic and treatment facilities for demyelinating disease in Indonesia, we do not have neurology services, nor imaging facilities, specifically dedicated to demyelinating diseases. Nationwide availability of diagnostic facilities is described in Table 4.

The treatment of patients with demyelinating diseases is generally covered by the national health insurance, with the exception of disease-modifying drugs. Serial MRI examinations are done at follow-up, usually every 6 to 12 months, depending on the clinical course of individual patients. Supportive medical care and rehabilitation for chronically ill or disabled patients are available, such as walking aids and physical therapy, covered by the national health insurance. However, further services such as assisted living are not covered and only affordable to select patients. It should also be noted that MS specialist nurses are still not available in Indonesia, which limits the option for specialized care, especially those requiring home visits.

In summary, we hope that this overview will lead to a better awareness of the spectrum of demyelinating diseases of the central nervous system in Indonesia. Recent research developments have redefined the broad range of clinical, biochemical, and pathological phenotypes of demyelination. It is a challenge for a country such as Indonesia to implement these scientific medical advances for the greater benefit of our patients, within the constraints of what is socially, technologically, economically, and logistically achievable.

## Figures and Tables

**Table 1:** MRI protocol comparison.

MAGNIMS MR protocol recommendation [10]	Standard MR protocol in our center
**Brain**	
*Baseline evaluation*	*Baseline and follow-up examinations*
Mandatory sequences	(i) Unenhanced axial and sagittal T1-weighted
(i) Axial proton-density and/or T2-FLAIR/T2-weighted	(ii) Contrast-enhanced axial, sagittal, and coronal T1-weighted
(ii) Sagittal 2D or 3D T2-FLAIR	(iii) Axial and coronal T2-weighted
(iii) 2D or 3D contrast-enhanced T1-weighted	(iv) Axial T2-FLAIR
Optional sequences	(v) Axial T2∗ gradient-echo (GRE)
(i) Unenhanced 2D or high-resolution isotropic 3D T1-weighted	(vi) Axial diffusion-weighted imaging
(ii) 2D and/or 3D dual inversion recovery	
(iii) Axial diffusion-weighted imaging	
*Follow-up examinations*	
Mandatory sequences	
(i) Axial proton-density and/or T2-FLAIR/T2-weighted highly recommended	
(ii) 2D or 3D contrast-enhanced T1-weighted	
Optional sequences	
(i) Unenhanced 2D or high-resolution isotropic 3D T1-weighted	
(ii) 2D and/or 3D dual inversion recovery	
(iii) Axial diffusion-weighted imaging	

**Spinal cord**	
*Sagittal imaging*	*Sagittal imaging*
Mandatory sequences	(i) Unenhanced T1-weighted
(i) Dual-echo (proton-density and T2-weighted) conventional and/or fast spin-echo	(ii) Contrast-enhanced T1-weighted with fat suppression
(ii) STIR (as an alternative to proton-density-weighted)	(iii) Conventional T2-weighted
(iii) Contrast-enhanced T1-weighted spin-echo (if T2 lesions present)	(iv) T2-weighted with turbo inversion recovery magnitude (TIRM)
Optional sequences	
(i) Phase-sensitive inversion recovery (as an alternative to STIR at the cervical segment)	
*Axial imaging*	*Axial imaging*
Optional sequences	(i) Unenhanced T1-weighted
(i) 2D and/or 3D T2-weighted fast spin-echo	(ii) Contrast-enhanced T1-weighted with fat suppression
(ii) Contrast-enhanced T1-weighted spin-echo	(iii) T2-weighted with turbo inversion recovery magnitude (TIRM)

**Table 2:** Ethnic group distributions of Indonesian MS and NMO patients.

Ethnic group (%)	MS	NMO
Javanese	36.4	22.2
Sundanese	9.1	5.5
Betawi	6.0	11.1
Batak	3.0	11.1
Minangkabau	12.1	5.5
Chinese descents	24.2	16.7
Others^∗^	9.2	27.9

^∗^Consists of Malay, Manado, Minahasa, Lampung, Palembang, Aceh, and Arab descents.

**Table 3:** Sex and age distribution of Indonesian MS, NMO, and ADEM patients.

	MS	NMO	ADEM
Sex (%)			
Male	18.2	11.1	78.6
Female	81.8	88.9	21.4
Median age (range) (year)	30 (20-61)	27 (18-64)	11 (1-38)

**Table 4:** Demyelinating disease diagnostic facility availability in Indonesia.

Diagnostic facility	Nationwide availability	National health insurance coverage status	Comments
MRI	195 units available in 25 of 34 provinces (with a ratio of 1 MRI unit to 1.3 million people)	Covered	Only single examination in one appointment is covered. Brain and spinal cord MRI cannot be performed in one appointment.
AQP4-IgG	3 facilities	Not covered	One facility is reserved for research purposes only. One facility is a private laboratory which sends the specimens to be examined in the United States.
CSF oligoclonal band	2 facilities	Not covered	One facility is a private laboratory which send the specimens to be examined in the United States.
MOG-IgG	1 facility	Not covered	Currently reserved for research purposes only.
VEP	444 units available in 33 of 34 provinces	Covered	—

## Data Availability

Access to data is restricted due to legal and ethical concerns.
